# The Principles Regulating the Natural Evacuation of Abscesses

**Published:** 1869-02

**Authors:** Samuel Logan

**Affiliations:** Professor of Surgery New Orleans School of Medicine, Late Prof. Anatomy Medical College of Virginia, etc.


### Selections.

The Principles Regulating the Natural Evacuation of Abscesses: By Samuel Logan, M. D., Professor of Surgery New Orleans School of Medicine, late Prof. Anatomy Medical College of Virginia, etc.—Without a careful examination of the facts, nothing seems more beautifully made out than the theory of the natural processes involved in the elimination of collections of pus from the living body. This theory is so familiar to our readers, that I need not describe it in detail. It is embodied in the proposition or law that the collection progresses in the direction where there is least resistance, and ultimately ulcerates its way through to the surface, which had been previously strangulated by the pressure of the abscess against the blood vessels which supply it. But does this theory explain all the cases in which abscesses work their way to the surface? It does not. Many cases occur where the collection passes in a direction in which the anatomical constituents indisputably present much more resistance than over many other portions of the periphery of the abscess. As instances in point, I may cite the frequent passage of such collections directly through the toughest layers of strong and inelastic fascial expansions, instead of dissecting their way up or down the limb, along the muscular intervals, or between the bones. These glaring violations of the alleged law have long since caused me to doubt its validity, and to seek some more comprehensive generalization by which the phenomena may be more rationally explained. I believe I have succeeded in finding one. Let it be understood, however, that I do not undertake to deny the existence of the influences heretofore recognized as those by which these purulent collections are brought to the surface. I intend only to affirm that there is a more potent law which controls those influences, and sometimes even directly and successfully oppose them. I claim that there is a higher generalization by which the phenomena can be more rationally explained.

The law I would announce may be couched in the following terms: *Abscesses, as a rule, must move in the same direction as the arterial blood current which mainly supplies their periphery and their so-called pyogenic membrane.* How this law works will be best explained by supposing a case of circumscribed abscess, and giving my views of the manner in which it progresses toward its spontaneous evacuation. Suppose an acute circumscribed abscess located just under the planter fascia, a deep "stone bruise," as it is vulgarly called. As such an abscess increases in size, it would tend, were its progress mainly dependent upon the degree of resistance met with at different points of its periphery, to move in almost any other direction than to the surface of the sole of the foot. But do we not often find such collections slowly and painfully working their way through the tough plantar fascia to this surface? How can this happen? The arterial branches which supply the periphery of such an abscess come from the two plantars. These lie deeper down than the abscess, and the branches which supply the periphery and walls of the collection pass consequently toward its deeper side. The result is, that on that side of the abscess the blood is carried by the finer arterioles uninterruptedly to the parts in that vicinity, and the radicles of the returning veins are subjected to pressure only at their termination. The result is, that the tissues on that side have their circulation comparatively intact even up to the plastic wall of the abscess itself. The nutrition of these parts is therefore but little disturbed, and we accordingly find that the wall of the abscess on that side is often seen to be decidedly thicker than on the side toward which the collection is moving; or, to express it in other terms, the nutrition of the parts on the *cardiac* is necessarily less disturbed than that of the parts on the *distal* side. The difference in the thickness of the plastic wall is the necessary result of this fact. But what is it that so materially interferes with the nutrition of the parts on the distal side? Most of the arteries which nourish that side *pass round the abscess, and the veins in returning are in like manner diverted from their natural course.* Both these vessels are therefore subjected to the lateral pressure of the abscess, the arteries, even before they have broken into their smaller branches, and the veins after their radicles have united into trunks. This condition of affairs necessarily leads to a most serious disturbance of the circulation, and a corresponding derangement of the nutrition of the distal parts. The arterial coats presenting greater resistance than the veins, whose walls are collapsed more readily, more blood is carried to the part by the former than can be readily returned by the latter; and thus we have a tendency to the characteristic congestive form of inflammation usually seen in such cases when the collection gets near the surface. The same causes exist when the abscess is too deep for inspection and we are therefore authorized in presuming that this form of hyperæmia or congestive inflammation precedes the abscess from the commencement of its progress. The œdema caused by the congestion further impairs the vitality of the parts; these become sodden, melt down, and are absorbed or slough off; and in this way even the toughest layers of fascia are made to yield, if they lie over the distal wall of the abscess. But it sometimes happens that, propelled at first by these causes, the abscess comes in time to the immediate proximity of a layer of tissue whose vascular supply is derived from arteries flowing in an opposite direction. These vessels and their minute venules not being cut off in the rear—so to express it—carry a comparatively uninterrupted supply of blood directly to the tissues opposing the abscess; their corresponding veins and venules, being but little interfered with, return the blood, and the circulation and nutrition of these tissues are, it may be, even more active than those on the other side of the abscess. The abscess is now arrested, and a conflict ensues, with a change in the after direction of the purulent collection. The result is, that the abscess takes a new direction latterly, this being again regulated by the preponderating direction of the arterial supply to the remaining portion of the periphery of the same. As an illustration of this conflict between two arterial currents, I may instance the remarkable manner in which certain structures protect themselves from destructive progress of purulent collections. For example, the larger veins and arteries nourished by their own vasa vasorum, will turn aside the advancing abscess; the muscular tissue, protected as by a shield, in virtue of its liberal supply of vessels, will most successfully avert the threatened danger; while on the other hand the hard unyielding bone too easily yields to the pressure of an abscess resting on its surface, for the circulation of its outer layers comes from the direction of the periosteum, the arterial current thus coinciding with the progress of the abscess, and therefore being the more readily cut off. Observe, too, in this last example, how the eroding process is more or less arrested as it approaches the interior of the bone. Here we find another arterial current coming from the opposite direction, *i. e.*, from the medullary vessels, and usually the progress of the abscess is here arrested. The same principles will be frequently found to apply to the elucidation of the so-called pressural effects of tumors. Take, for example, the case of an aortic aneurism resting against the spinal column, and excavating a bed for itself in the bodies of one or more vertebræ. If pressure alone can account for the absorption of the boney tissue in such a case, the *softer* of the two opposing surfaces should be the one to yield. But the bone and not the tumor becomes indented. The blood vessels supplying the outer layers of bone are cut off by the pressure of the tumor against the periosteum through which they pass, and the loss of nutritive activity in the bone is the result and the absorption follows. But this absorption is seldom, if ever, allowed to progress beyond a certain depth, and that limit is reached as soon as the tumor reaches the strata of bone whose nutrition depends on the liberal supply of blood furnished by the branches of the thecal vessels coming from the opposite direction as they do. Even in the oldest aneurisms we never find the whole thickness of the vertebræ destroyed. I have never seen or read of such a case. In fact, the application of this principal to all the clinical facts susceptible of explanation by it would take more space and time than is at present at my command, and I leave to the reader the further consideration of the subject in its suggestive details.

